# Mutation of the Protein Kinase C Site in Borna Disease Virus Phosphoprotein Abrogates Viral Interference with Neuronal Signaling and Restores Normal Synaptic Activity

**DOI:** 10.1371/journal.ppat.1000425

**Published:** 2009-05-08

**Authors:** Christine M. A. Prat, Sonja Schmid, Fanny Farrugia, Nicolas Cenac, Gwendal Le Masson, Martin Schwemmle, Daniel Gonzalez-Dunia

**Affiliations:** 1 INSERM, U563, Centre de Physiopathologie de Toulouse Purpan and Université Paul-Sabatier, Toulouse, France; 2 Department of Virology, University of Freiburg, Freiburg, Germany; 3 INSERM U862, Université Bordeaux 2, Bordeaux, France; 4 Avenir Team, INSERM U563, Centre de Physiopathologie de Toulouse Purpan and Université Paul-Sabatier, Toulouse, France; University of California San Francisco, United States of America

## Abstract

Understanding the pathogenesis of infection by neurotropic viruses represents a major challenge and may improve our knowledge of many human neurological diseases for which viruses are thought to play a role. Borna disease virus (BDV) represents an attractive model system to analyze the molecular mechanisms whereby a virus can persist in the central nervous system (CNS) and lead to altered brain function, in the absence of overt cytolysis or inflammation. Recently, we showed that BDV selectively impairs neuronal plasticity through interfering with protein kinase C (PKC)–dependent signaling in neurons. Here, we tested the hypothesis that BDV phosphoprotein (P) may serve as a PKC decoy substrate when expressed in neurons, resulting in an interference with PKC-dependent signaling and impaired neuronal activity. By using a recombinant BDV with mutated PKC phosphorylation site on P, we demonstrate the central role of this protein in BDV pathogenesis. We first showed that the kinetics of dissemination of this recombinant virus was strongly delayed, suggesting that phosphorylation of P by PKC is required for optimal viral spread in neurons. Moreover, neurons infected with this mutant virus exhibited a normal pattern of phosphorylation of the PKC endogenous substrates MARCKS and SNAP-25. Finally, activity-dependent modulation of synaptic activity was restored, as assessed by measuring calcium dynamics in response to depolarization and the electrical properties of neuronal networks grown on microelectrode arrays. Therefore, preventing P phosphorylation by PKC abolishes viral interference with neuronal activity in response to stimulation. Our findings illustrate a novel example of viral interference with a differentiated neuronal function, mainly through competition with the PKC signaling pathway. In addition, we provide the first evidence that a viral protein can specifically interfere with stimulus-induced synaptic plasticity in neurons.

## Author Summary

Neurotropic viruses have evolved diverse strategies to persist in their host, with variable consequences for brain function. The investigation of these mechanisms of persistence and associated disease represent a major issue in viral pathogenesis, as it may also improve our understanding of human neurological diseases of unclear etiology for which viruses are thought to play a role. In this study, we have examined the mechanisms whereby the neurotropic Borna disease virus (BDV) can selectively interfere with synaptic plasticity upon infection of neurons. Using genetically engineered recombinant viruses, we show that the phosphorylation of BDV phosphoprotein (P) by the cellular protein kinase C (PKC) is the main determinant for this interference, mainly by competing with the phosphorylation of the natural PKC substrates in neurons. A mutant virus in which the PKC phosphorylation site of P has been destroyed no longer interferes with this signaling pathway. As a result, the calcium dynamics and electrical activity in response to stimulation of neurons infected with this mutant virus are completely corrected and become similar to that of non-infected neurons. Thus, our findings uncover a previously undescribed mechanism whereby a viral protein interferes with neuronal response to stimulation.

## Introduction

The finding that persistent viruses could selectively affect differentiated functions of their target cell without causing cell lysis or widespread inflammation was first demonstrated more than 25 years ago [1]. This type of viral persistence, characterized by minimal cell damage, seems particularly well suited for the central nervous system (CNS) given the limited capacity of renewal of CNS resident cells, in particular of neurons. Viral interference with selected signaling pathways will nevertheless disrupt cellular homeostasis and cause disease [2]. As viral impairment of neurons may lead to behavioral or cognitive impairment, it was therefore hypothesized that persistent viruses could play a role in human mental disorders of unclear etiology [3],[4]. To date, the mechanisms whereby viruses can interfere with brain function are not well understood and are strongly dependent on the strategy that a given virus has developed to persist in the CNS [5],[6]. For viruses actively replicating in neuronal cells, one hypothesis is that the expression and/or accumulation of viral products in the cell may affect neuronal activity and cause disease. To date, it is clear that much is needed for a better understanding of the pathogenesis of persistent viral infections of the CNS and for the identification of the viral determinants responsible for the associated diseases.

Borna disease virus (BDV) is a highly neurotropic, non-cytolytic virus that provides an ideal paradigm for studying the behavioral correlates of CNS viral infections. BDV is an enveloped virus with a non-segmented, negative strand RNA genome [7],[8]. In contrast to other *Mononegavirales*, BDV replicates in the nucleus of infected cells [9] and uses the host cell splicing machinery for maturation of viral transcripts [10],[11]. The BDV compact genome encodes for six proteins, namely, the nucleoprotein (N), phosphoprotein (P), protein X, matrix protein (M), glycoprotein (G), and polymerase (L). Whereas M and G are involved in particle formation, P, N, L, and X are components of the polymerase complex. BDV infects a wide variety of mammals [12],[13] and is associated with a large spectrum of neurological disorders, ranging from immune-mediated diseases to behavioral alterations without inflammation [12],[14],[15]. These disorders are reminiscent of symptoms observed in certain human neuropsychiatric diseases [16]. Evidence suggest that BDV infections may also occur in humans, although a link between BDV infection and any human neurological disease has not been firmly established yet [17]–[19]. The neurobehavioral manifestations associated with BDV infections in animals are partly due to the selective tropism of BDV in the CNS for neurons of the cortex and hippocampus [15],[20],[21], which govern many cognitive and behavioral functions [22].

In an effort to better characterize the impact of BDV persistence on neuronal function, we recently analyzed the neuronal activity of primary cultures of neurons infected with BDV, using both functional imaging and electrophysiological approaches [23],[24]. These studies clearly showed that BDV interferes with activity-dependent plasticity, while leaving the basal properties of neuronal activity unaffected. Moreover, the selective impairment of neuronal plasticity due to BDV infection was correlated to a reduced phosphorylation of the neuronal targets of Protein Kinase C (PKC), a kinase that plays important roles in the regulation of neuronal activity [25]. Amongst the different viral proteins, BDV P appeared as the most plausible candidate for mediating this interference. Similar to the phosphoproteins of other *Mononegavirales*, BDV P is a component of the viral polymerase complex, which serves several functions in the viral life cycle. These functions are thought to be regulated, at least in part, by its phosphorylation by cellular kinases [26]. BDV P is preferentially phosphorylated at serine residues 26 and 28 by PKC and, to a lesser extent, at serine residues 70 and 86 by casein kinase II (CKII) [27]. Taken together, these observations led us to postulate that BDV P may serve as a PKC kinase decoy substrate when expressed in neurons, resulting in the decreased phosphorylation of other PKC neuronal targets. Although transfection experiments using a BDV P expressing plasmid provided the first evidence that this could indeed be the case [24], a formal demonstration of the role of BDV P in PKC-dependent signaling and on neuronal activity was needed.

The newly established reverse genetics technique allowing the generation of recombinant BDV (rBDV) entirely from cDNA has provided means to test this hypothesis directly [28]. Recently, we characterized rBDV expressing P mutants lacking either the PKC or the CKII phosphorylation sites, upon replacement of the corresponding serine residues with alanines to abrogate phosphorylation [29]. We showed that phosphorylation of BDV P acts as a negative regulator of the viral polymerase complex activity, in contrast to what has been shown for other *Mononegavirales*. Here, we infected primary cultures of rat hippocampal and cortical neurons with these different recombinant viruses and analyzed their phenotype and responses to stimulation. Using a rBDV where P can no longer be phosphorylated by PKC, we demonstrate a complete suppression of viral interference with neuronal activity. This was shown not only at the molecular level in terms of PKC-dependent signaling, but also at the functional level by studying calcium responsiveness to depolarization and the electrophysiological network properties of cultured neurons.

Thus, our findings illustrate a novel example of viral interference with a differentiated neuronal function, mainly through competition with the PKC signaling pathway. In addition, we provide the first evidence that a viral protein can specifically interfere with stimulus-induced synaptic plasticity in neurons.

## Results

### Characterization of the mutant viruses in primary hippocampal neuronal cultures

To study the impact of BDV P phosphorylation on viral neuropathogenesis, we used a recombinant BDV in which the two serine (S) residues in position 26 and 28 of P have been replaced by alanine (A) residues (rBDV-AASS). We have previously shown that these mutations, when introduced using the recently established technique for generating BDV from cDNA [28], completely abolishes BDV P phosphorylation by PKC, while still supporting full polymerase activity and viral growth [27],[29]. As a control, we also used a recombinant wild-type BDV (rBDV-wt), composed of the canonical sequence of wild-type BDV strain He/80 [30].

**Figure 1:**
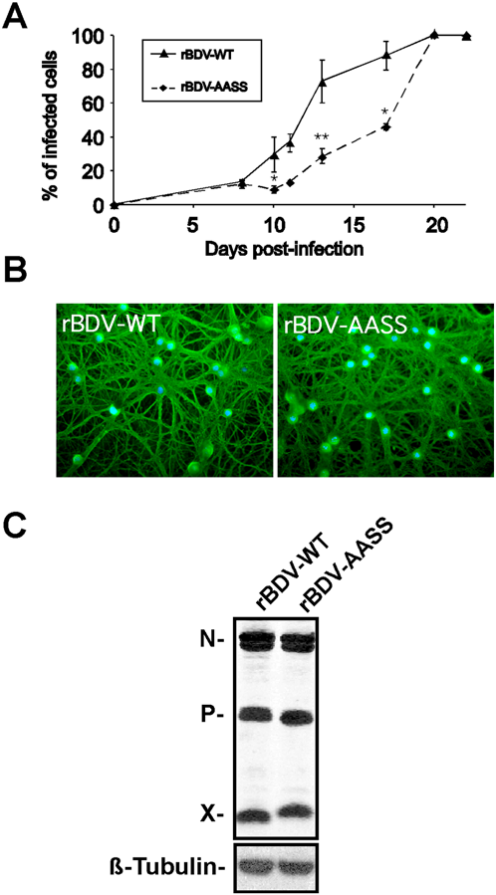
Characteristics of rBDV infection of primary cultures of hippocampal neurons. (A) Quantification of virus spread. On different days of culture, percentages of BDV positive neurons were assessed by immunofluorescence using an N-specific antiserum, relative to the total number of nuclei. This measure was carried out on two replicate wells for each time point, after random selection of five different fields for each replicate. Results are expressed as mean±s.e.m. of four independent experiments. *, p<0.05; **, p<0.01 by unpaired *t*-test. (B) Immunofluorescence analysis of neurons infected with the rBDV-WT and rBDV-AASS viruses, 21 days post-infection. BDV-N was detected with a rabbit polyclonal antibody, followed by a FITC-labeled secondary antibody (green), while nuclei are stained with DAPI (blue). Original magnification, ×200. (C) Western blot analysis of neuronal extracts from cultures infected the rBDV-WT and rBDV-AASS viruses, 21 days post-infection, using antibodies specific for the BDV N, P and X proteins. ß-Tubulin was detected as a control reaction for equal loading of each lane. Results are representative of four independent experiments.

We first analyzed the impact of these mutations on the kinetics of viral spread in primary hippocampal rat neuronal cultures. To this end, we infected neuron-rich cultures (>80% neurons) one day after their plating with 300 focus forming units (FFU) per well of either rBDV-wt or rBDV-AASS cell-released virus (prepared from persistently infected Vero cells). We studied viral dissemination occurring after primary infection by assessing expression of the BDV nucleoprotein at different times post-infection, using immunofluorescence microscopy to quantify viral spread. Consistent with our previous reports using wild-type BDV [31], infection with rBDV-wt was apparent by 5 to 7 days post-infection, with a low percentage (<10%) of all neurons being positive for BDV nucleoprotein. The virus spread rapidly increased thereafter and by day 20, more than 95% of neurons were infected (Figure 1A). In contrast, spread of the rBDV-AASS virus was significantly delayed, in particular between days 10 and 17 post-infection, a time when viral dissemination speed is usually maximal due to the increased density of the neuronal network [31]. Thus, the phosphorylation of BDV P seems to be required for optimal transneuronal virus spread in primary neurons.

Importantly, despite the delayed kinetics for rBDV-AASS, infection ultimately disseminated to the whole neuronal cultures. By 20–21 days post-infection, the large majority of neurons was positive for BDV antigens, with no significant differences between cultures infected with either rBDV-wt or rBDV-AASS, as assessed by immunofluorescence analysis (Figure 1B). At this stage, quantitative Western blot analysis revealed that comparable amounts of N, P and X viral proteins were present in neurons infected with both rBDV-wt and rBDV-AASS viruses. Consistent with our previous characterization of rBDV-AASS, we observed a delayed migration for the X protein [29], presumably resulting from the two amino acids substitutions introduced in X when generating the rBDV-AASS mutant.

In conclusion, both recombinant viruses were able to infect the totality of our neuronal cultures, albeit with a delayed kinetics for rBDV-AASS. For our subsequent signaling and functional studies, we therefore used neurons that had been infected for at least 21 days and verified for each experiment that infection was indeed complete prior to any subsequent analysis.

### The S26/28A mutation of BDV P corrects the interference with PKC signaling

**Figure 2:**
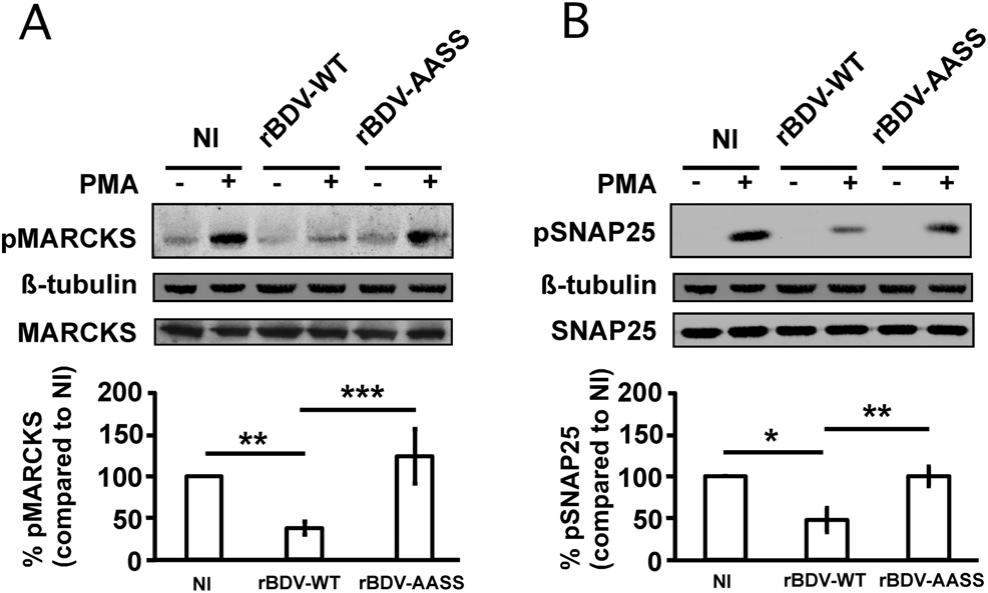
Analysis of PKC signaling in hippocampal neurons infected with the different recombinant viruses. Total extracts were prepared from non-infected (NI) neurons and neurons infected with the rBDV-WT or rBDV-AASS viruses (21 days post-infection), after stimulation or not with 1 µM PMA for 10 min. Equivalent protein amounts were analyzed by Western blot with specific antibodies for (A) phospho-MARCKS (pMARCKS; PKC site at Ser152/156) and (B) phospho-SNAP-25 (pSNAP-25, PKC site at Ser187). ß-tubulin and total MARCKS and SNAP-25 were used to normalize expression. Bottom graphs show the fluorometric quantification, using the Odyssey imager, of four to ten independent experiments. Quantification results were expressed as percentage of increase relative to the response of NI neurons, which was set at 100%. Values are expressed as mean±s.e.m. *, p<0.05; **, p<0.01; ***, p<0.001 by unpaired *t-*test.

Recently, we have shown that BDV-induced impairment of potentiation of synaptic activity was due to an interference with the PKC-dependent phosphorylation of synaptic proteins that modulate neuronal activity [24]. To determine the impact of the destruction of the PKC phosphorylation sites on BDV P, we directly stimulated the PKC pathway of neurons using the phorbol ester PMA and analyzed the phosphorylation status of two major PKC targets in neurons, myristoylated alanine-rich C kinase substrate (MARCKS) and synaptosomal-associated protein of 25 kDa (SNAP25) [32],[33]. Consistent with our previous reports, we showed by quantitative Western blot analysis that the phosphorylation of these two neuronal PKC targets was significantly impaired in neurons infected with rBDV-wt (Figure 2A and 2B). In contrast, there was a complete restoration of phospho-MARCKS and phospho-SNAP25 levels in neurons infected with rBDV-AASS, with levels being comparable to those observed in non-infected neurons following PKC stimulation.

### Analysis of calcium responsiveness to depolarization in neurons infected with rBDV-wt and rBDV-AASS

**Figure 3:**
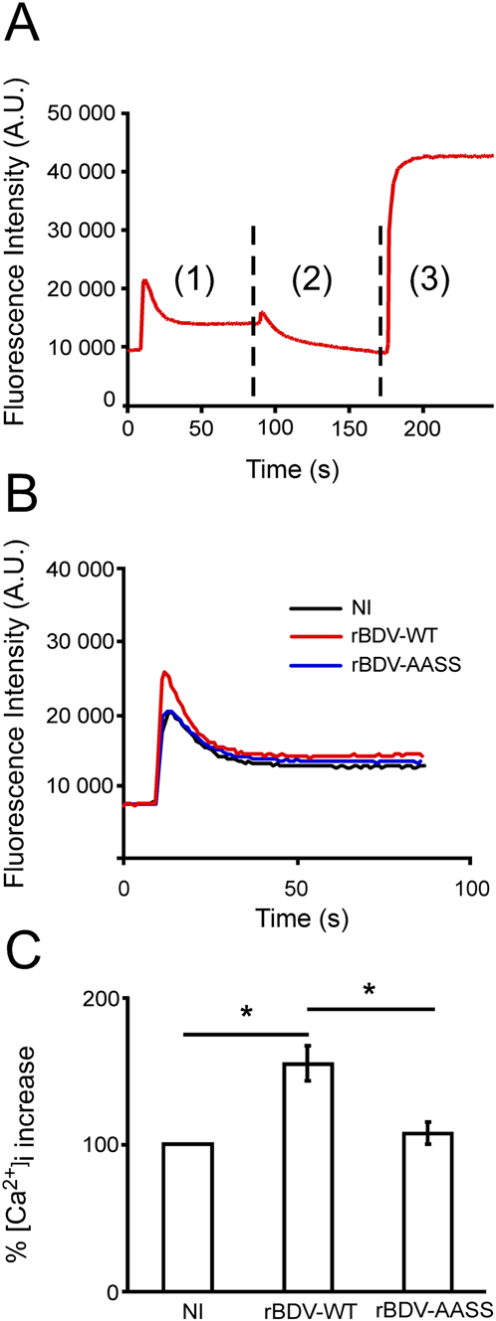
Calcium responses recorded from hippocampal neurons infected with the rBDV-WT and rBDV-AASS viruses. (A) Representative example of a time-course measurement of free intracellular calcium by microspectrofluorimetry. Fluo-3 loaded neuronal cultures were recorded over a 250 second-period (1 measure per second) upon (1) exposure to 50 mM KCl; (2) treatment with thapsigargin/ionomycin in the presence of 10 µM EGTA to record the minimum fluorescence; (3) stimulation with 120 mM CaCl_2_ (a concentration sufficient to chelate EGTA), allowing the recording of the maximum fluorescence. (B) Representative example of the peak calcium responses following depolarization with 50 mM KCl of control neurons (NI) and neurons infected with rBDV-WT or rBDV-AASS viruses. Data were plotted on the same graph for comparison. (C) Quantitative analysis of peak calcium responses. In each case, recordings were performed on six separate wells, and data represent the mean of four independent experiments. [Ca^2+^]_i_ increase was expressed as the increase in fluorescence signal after stimulation relative to the increase measured in NI neurons, which was set at 100%. *, p<0.05 by unpaired *t*-test.

SNAP25 is a synaptic protein that plays an essential role in neurotransmitter release through regulation of synaptic vesicle exocytosis [34]. In addition, SNAP-25 also modulates calcium dynamics in response to depolarization by acting on Voltage-Gated Calcium Channels (VGCC). It has recently been shown that activity-dependent phosphorylation of SNAP-25 is mediated by PKC and is required for negative regulation of VGCCs [35]. Indeed, PKC phosphorylation of SNAP-25, by promoting inhibition of VGCCs decreases calcium signaling and controls neuronal excitability. This led us to investigate whether the differences observed between neurons infected with rBDV-wt and rBDV-AASS in the phosphorylation of SNAP25 would have an impact on their calcium responsiveness upon depolarization. We therefore analyzed the kinetics of calcium changes in response to depolarization after exposure of infected and control neurons to 50 mM KCl. Infection of neurons with rBDV-wt significantly enhanced their response to depolarization, with a peak calcium response being about fifty percent stronger than control non-infected neurons. In sharp contrast, the calcium response of neurons infected with rBDV-AASS was similar to that of non-infected neurons (Figure 3A and 3B). Together, these findings suggest that BDV mediated interference with PKC-dependent phosphorylation, by reducing phospho-SNAP-25 levels, leads to calcium hyper-responsiveness and that mutation of the BDV P phosphorylation sites restores normal calcium responses.

### Mutations of the overlapping X protein in rBDV-AASS are not involved in viral interference with PKC-dependent signaling

**Figure 4:**
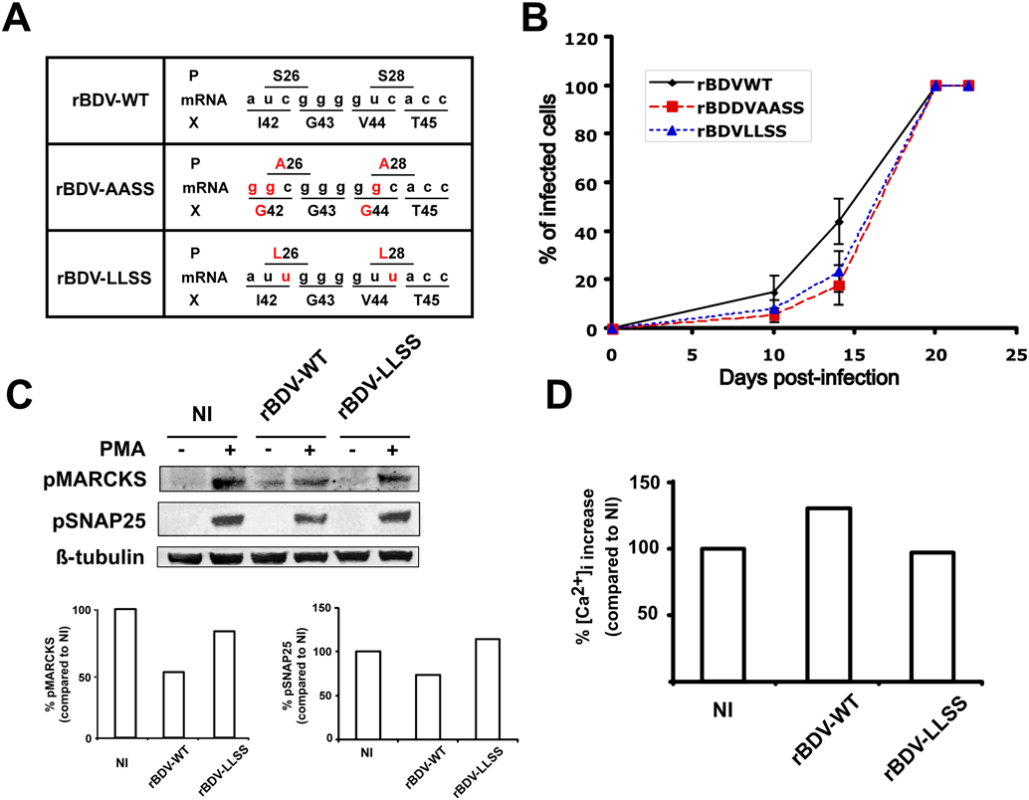
Phenotypic characterization of a recombinant BDV (RBDV-LLSS) bearing mutations in the PKC phosphorylation site of P (S26L;S28L), but none in the overlapping X protein. (A) Cartoon depicting part of the P and X overlapping open reading frames of the 0.8 kb mRNA from the indicated viruses and their translation products. Amino acids and nucleotides highlighted in red indicate differences from the wild-type situation. (B) Comparative analysis of virus spread for rBDV-wt, rBDV-AASS and rBDV-LLSS recombinant viruses. Percentages of BDV positive neurons were assessed by immunofluorescence on different days of culture, as described in Figure 1. This measure was carried out on two replicate wells for each time point, after random selection of five different fields for each replicate. Results are expressed as mean±s.e.m. of three independent experiments. (C) Analysis of PKC signaling in hippocampal neurons. Total extracts were prepared from non-infected (NI) neurons and neurons infected with the rBDV-WT or rBDV-LLSS viruses (21 days post-infection), after stimulation or not with 1 µM PMA for 10 min. Equivalent protein amounts were analyzed by Western blot with specific antibodies for phospho-MARCKS and phospho-SNAP-25. ß-tubulin was used to normalize expression. Bottom graphs show the fluorometric quantification, using the Odyssey imager, of one representative experiment out of three. Quantification results were expressed as percentage of increase relative to the response of NI neurons, which was set at 100%. (D) Quantitative analysis of peak calcium responses recorded from hippocampal neurons infected with the rBDV-WT and rBDV-LLSS viruses. In each case, recordings were performed on six separate wells, and data represent results of a representative experiment out of two. [Ca2+]i increase was expressed as the increase in fluorescence signal after stimulation relative to the increase measured in control neurons, which was set at 100%.

Since P is expressed from a bicistronic mRNA encoding P and X, the introduced mutation into the open reading frame of P in the rBDV-AASS mutant also resulted in two amino acid substitutions in the X protein (Figure 4A). We previously showed that these mutations had no impact on X binding efficiency to P or its ability to interfere with the polymerase activity [29]. However, we wanted to exclude formally the possible contribution of the X mutations in the phenotype of the rBDV-AASS mutant. To explore the importance of BDV-P phosphorylation in the presence of wild-type X, we generated a new recombinant BDV, in which S26 and S28 of BDV P were substituted with leucine residues (rBDV-LLSS, Figure 4A). Similar to rBDV-AASS, viral spread of rBDV-LLSS was delayed upon infection of hippocampal neurons (Figure 4B). Likewise, we also demonstrated the restoration of phospho-MARCKS and phospho-SNAP25 levels in neurons infected with rBDV-LLSS, with levels being comparable to those observed in non-infected neurons following PKC stimulation (Figure 4C). Finally, the calcium hyper-responsiveness observed in rBDV-wt infected neurons was also corrected in neurons infected with rBDV-LLSS (Figure 4D). Thus, the phenotype observed with rBDV-LLSS is very similar to rBDV-AASS, providing additional evidence that our findings are indeed due to phosphorylation of BDV-P by PKC and not to the mutations present in X.

**Figure 5:**
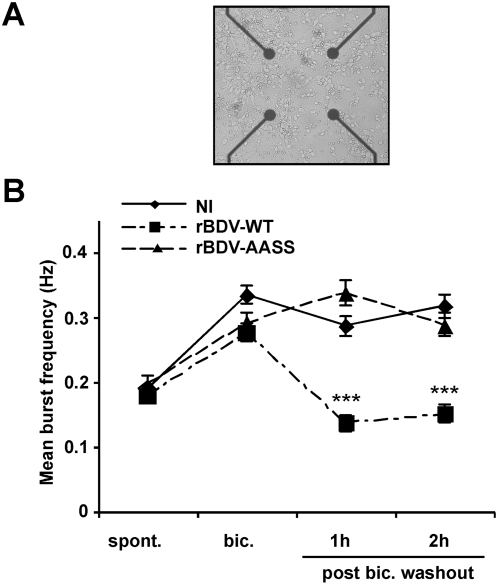
Analysis of the electrophysiological properties of neurons infected with the different recombinant viruses. (A) Representative view of cortical neurons cultured in an MEA dish. Electrodes are spaced by 200 µm; electrode diameter is 30 µm. Original magnification, ×50. (B) Quantitative analysis of the mean burst frequency for non-infected (NI) neurons and neurons infected with the rBDV-WT or rBDV-AASS viruses (21 days post-infection). The mean burst frequency was calculated under spontaneous conditions (spont), bicuculline stimulation (bic), as well as 1 h and 2 h after bicuculline washout. For each condition, data were acquired from 170 to 311 electrodes, from four independent experiments using four MEA dishes for each mock or virus infection. Values are expressed as mean±s.e.m. ***, p<0.001 by unpaired *t*-test.

The S26/S28A mutation of BDV P corrects the defects in electrical activity observed in rBDV-wt infected neurons. Given the dramatic consequences of the S26/28A mutation on neuronal signaling and calcium responsiveness, we next studied its impact on the electrophysiological properties of infected neurons. Recently, we described a cell culture system using microelectrode arrays (MEA), which allows to monitor the firing pattern of a neuronal network grown on a grid of sixty electrodes embedded in a culture dish (Figure 5A) [36],[37]. Using this system, we showed that BDV selectively blocks activity-dependent enhancement of neuronal network activity, one form of synaptic plasticity thought to be important for learning and memory [23],[38]. Given the central role of PKC in synaptic plasticity, we hypothesized that this defect could be linked to BDV interference with this signaling pathway. To test this hypothesis, we compared the electrophysiological properties of cultures of cortical neurons infected with rBDV-wt or rBDV-AASS viruses, using the MEA culture system. All experiments were again performed at day 21, to allow spreading of both viruses to the totality of the MEA cultures. At this time point, neurons have developed a rich network of processes and form numerous functional synaptic contacts [31],[39]. In agreement with our previous results [23], we did not observe any significant difference in the spontaneous network firing activity between neurons infected with either rBDV-wt or rBDV-AASS viruses (Figure 5B). This firing pattern was also indistinguishable from that of control non-infected neurons. Next, we induced increased synaptic efficacy by exposing neuronal cultures for 15 min to 50 µM of bicuculline, a GABA_A_ receptor antagonist. Treatment with this antagonist leads to the removal of the tonic inhibition imposed by GABAergic interneurons on the network [40]. As a result, we observed a significant increase of the mean burst frequency, which shifted from 0.175 Hz to 0.285–0.31 Hz (Figure 5B). Here again, the behavior of all neuronal cultures was remarkably similar, regardless of their infection status. Interestingly, the increase in the strength of the synaptic connections triggered by bicuculline lasts for several hours upon removal of the drug [40],[41] and is thought to represent the cellular basis of learning and memory [38]. In non-infected neurons, we indeed observed this maintenance of a high level of network activity, lasting more than two hours after washout of the drug. In contrast, neurons infected with rBDV-wt had returned to basal levels of network activity already one hour after bicuculline washout, confirming our previous results using wild-type BDV [23]. Very strikingly, the network properties of neurons infected with rBDV-AASS were completely different, as we observed the maintenance of high levels of synaptic activity persisting up to two hours after bicuculline washout, similarly to non-infected neurons. Therefore, a recombinant BDV which can no longer be phosphorylated by PKC on its mutated P protein has lost its capacity to block activity-induced synaptic potentiation.

## Discussion

**Figure 6:**
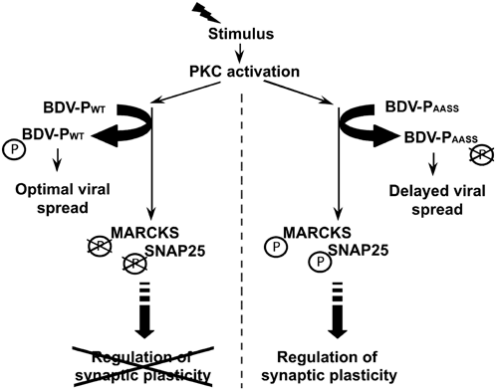
A model of P-mediated interference with PKC-dependent signaling and neuronal plasticity. Diverse potentiation stimuli will trigger activation of many kinases, including PKC. In cells infected with rBDV-WT (left), this will lead to enhanced phosphorylation of P, optimal viral spread and concomitant decreased activation of endogenous PKC substrates (among which MARCKS and SNAP-25), ultimately leading to impairment on neuronal plasticity. In neurons infected with rBDV-AASS (right), mutated P does not interfere with PKC-dependent signaling and neuronal plasticity is restored. However, lack of phosphorylation of P leads to viral attenuation.

The goal of our study was to provide further information about the mechanisms whereby BDV infection of neurons selectively interferes with synaptic plasticity and to identify the viral determinant responsible for this interference. Using a recombinant virus in which the PKC phosphorylation sites of BDV P protein have been destroyed, we demonstrate that primary cultures of neurons infected with this recombinant virus exhibit a behavior that becomes indistinguishable in many aspects to that of control, non-infected neurons. Therefore, our results clearly establish that BDV interference with PKC signaling, but also with calcium responses to depolarization and with network electrical properties, all result from the competition mediated by P with the phosphorylation of endogenous PKC substrates in neurons. We therefore propose a pathogenesis mechanism by which BDV would use PKC-dependent phosphorylation of P for its optimal spread in neurons, at the expense of an impaired response to potentiation stimuli of the infected neurons (Figure 6). Our findings provide strong evidence for a novel mechanism whereby a viral protein selectively blocks neuronal plasticity, representing a fascinating aspect of viral interference with neuronal functioning.

Interestingly, both mutant viruses rBDV-AASS and rBDV-LLSS were strongly delayed in its capacity to spread within the neuronal cultures. These findings suggest that PKC-dependent phosphorylation of P is an important parameter for efficient transmission of BDV from neuron to neuron. This may also explain the preferential BDV infection of CNS regions where PKC activity is high, such as hippocampus [42]. The underlying mechanism for this delayed spread is unclear and it is presently not possible to discriminate whether it is due to reduced cell-to-cell transmission or to a reduced capacity of the virus to be transported along neuronal processes. It does not seem to result from a decreased efficacy of viral replication, since analysis of the steady state levels of viral transcripts in rBDV-AASS- as well as in rBDV-LLSS-infected cells appeared to be normal [29]. It could be due to a less efficient transport of BDV ribonucleoparticles along the neuronal processes, resulting from impaired interaction of non-phosphorylated P with yet unidentified neuronal motor proteins involved in BDV trans-neuronal spread. Alternatively, the delayed spread may be a consequence of impaired virus assembly or release, similar to human respiratory syncytial virus, where phosphorylation of P regulates the viral budding process by blocking the interaction of P with the viral matrix protein [43]. Finally, mutations present in the X protein should also be taken into account, as we cannot exclude the possibility that these mutations may affect unknown functions of X, leading to impaired viral spread. However, this latter possibility seems unlikely as the rBDV-LLSS mutant, which has no mutation in X, also exhibits delayed spread kinetics in neurons.

Infection with the rBDV-AASS virus led to a complete restoration of the PKC-dependent phosphorylation of two neuronal targets that play crucial roles in modulating neuronal plasticity. Indeed, phosphorylation of MARCKS by PKC is implicated in actin-dependent cytoskeletal plasticity [44] and in the maintenance of long-term potentiation *in vivo* [45]. SNAP-25 is not only central for neuronal exocytosis but also for the regulation of calcium responsiveness. Modulation of neuronal excitability by SNAP-25, which is dependent on PKC, is thought to have crucial consequence for brain functioning [34]. The calcium hyper-excitability in response to depolarization that we observed after infection with rBDV-wt is consistent with a recent study showing that non-phosphorylable (S187A) SNAP-25 mutants also display increased calcium responsiveness [35]. Moreover, SNAP-25 S187A mutant mice exhibit behavioral abnormalities and hyperlocomotor activity, similar to what has been described following BDV infection [46]. Finally, genetic studies have demonstrated an association of polymorphisms in the human SNAP-25 gene with attention deficit hyperactivity disorder or cognitive performance [47],[48]. Nevertheless, as BDV is likely to affect all PKC neuronal substrates in the infected neurons, the relative contribution of the decreased phosphorylation of each of these substrates, including SNAP-25, is difficult to appreciate.

Very striking was the impact on the neuronal electrical activity measured using the MEA system. Since neurons infected with the rBDV-AASS mutant displayed a response to bicuculline stimulation that became indistinguishable from that of non-infected neurons, it is likely that interference with PKC signaling is indeed a main determinant for BDV impairment of neuronal functioning. The question remains open of whether the impairment of neuronal activity due to BDV infection is solely dependent on PKC inhibition. Using a global proteomic approach, we recently demonstrated that other neuronal pathways were altered by BDV infection, even prior to any stimulation [49]. At present, the link between some of these pathways, such as chromatin dynamics or transcriptional regulation and PKC-dependent signaling is not clear. Using infection with rBDV-AASS, in which PKC interference is suppressed, will be instrumental to test whether these other pathways are still affected, allowing to gain further insight on the relationship between the different BDV targets in neurons.

Phosphoproteins of non-segmented negative-strand RNA viruses are subunits of the viral polymerase complex and are all phosphorylated by host kinases [26]. Although many studies have addressed the role of P phosphorylation on the virus life cycle, very few have tested its consequences on the physiology of the target cell. In theory, other viral phosphoproteins could also block endogenous phosphorylation, including that of PKC. For example, it has been shown that rabies P can be phosphorylated by PKC [50]. However, BDV represents a unique example where neurons can be persistently infected with strong antigenic load and no widespread cytolysis. Thus, a possible interference with PKC-dependent phosphorylation in neurons becomes apparent following BDV infection due to its outstanding non-cytolytic replication strategy, whereas it would not be detected with other viral systems that kill their target cell within a few days.

As for the other *Mononegavirales*, BDV P is a multifunctional protein, which plays many roles in the virus life cycle. Besides its interference with PKC signaling, P has been reported to influence cellular functions at different levels. In particular, recent studies revealed interactions of P with a neurite outgrowth factor, amphoterin/HMG-1 [51], with the Traf family member-associated NF-κB (TANK)-binding kinase-1 (TBK-1) [52] and the gamma-aminobutyric acid receptor-associated protein [53]. Since these studies were based on transient transfection using non-neuronal cells, the relevance of these findings for BDV pathogenesis remains elusive and awaits further confirmation. It has also been shown that the expression of P in glial cells of transgenic mice leads to behavioral abnormalities [54], although the underlying mechanism was not identified. Given the important role of astrocytes in the regulation of neuronal activity [55], one hypothesis could be that P could also interfere with PKC-signaling in astrocytes and thereby disrupt glia-neuron communication. Interestingly, MARCKS, the main PKC substrate, is also expressed in astrocytes [56]. Finally, the two phospho-serine residues of P in themselves could have other unknown effects that could contribute to the phenotype observed, besides acting as PKC decoy substrates. Although none of the other known functions of P were affected, there may still be other uncharacterized functions of P that may be involved in the regulation of neuronal activity.

To date, the analysis of the mechanisms underlying viral interference with neuron-specific differentiated functions, particularly those that support synaptic activity has been hampered by the lack of suitable model systems and easily testable hypotheses. BDV infection has provided considerable new insight on these issues, and the recent availability of a reverse genetics system has offered a powerful tool to assess directly the role of individual viral proteins in virus-host interplay and pathogenicity. Our data unambiguously demonstrate the role of P as a decoy substrate interfering with PKC signaling pathway, a kinase which plays important roles in learning and behavior [25]. Moreover, they reveal an original strategy for a neurotropic persistent virus and provide clues to better understand the basis of neuronal impairment caused by BDV. It will be important in the future to test the impact of BDV P in vivo, either using animal models for BDV infection or expressing the wild-type and mutant forms of P in selected brain areas.

## Materials and Methods

### Primary culture of neurons and viral infections

Hippocampal neurons were prepared from newborn Sprague-Dawley rats and maintained in Neurobasal medium (Invitrogen, Cergy-Pontoise, France) supplemented with 0.5 mM glutamine, 1% fetal calf serum, 1% Penicillin/Streptomycin and 2% B-27 supplement (Invitrogen), as described [31],[57]. Neuronal cultures contained more than 80% neurons, as assessed by staining with the neuron-specific markers MAP-2 or ß-III Tubulin (data not shown). Neurons were infected one day after plating with cell-free BDV. Cell-released virus stocks were prepared as described [31],[57], using Vero cells persistently infected with the different recombinant viruses. BDV infection of neurons was verified by immunofluorescence for each experiment.

### Antibodies and reagents

We used: mouse monoclonal antibodies to SNAP25 (Synaptic Systems, Goettingen, Germany), ß-tubulin (Sigma-Aldrich, Lyon, France), rabbit polyclonals to phospho-MARCKS (Ser152/156, a site specifically phosphorylated by PKC; Cell Signaling Technology, Danvers, Massachusetts, USA), MARCKS (Chemicon-Millipore, Saint-Quentin-en-Yvelines, France). Phospho-SNAP25 (Ser187) antibody, a site specific for PKC phosphorylation [33] was kindly provided by Pr. M. Takahashi (Kitasato-University School of Medicine, Kitasato, Japan). All other antibodies have been described elsewhere [57]. Pharmacological agents were used at the following final concentrations: 1 µM PMA, 1 mM tetrodotoxin (TTX; Sigma-Aldrich), 100 mM of NMDA receptor blocker D-(-)-2-amino-5-phosphopentanoic acid (APV) and 40 mM of AMPA/kainate receptor blocker 6-cyano-7-nitroquinoxaline-2,3-dione disodium (CNQX; Tocris Bioscience, Bristol, United Kingdom). Bicuculline (Bicuculline methiodide, Tocris Biosciences) was used at a final concentration of 50 µM.

### Plasmid construction

To introduce point mutations into the P gene of the full-length BDV genome (pBDV-LLSS), assembly PCR using the plasmid pBRPolII-HrBDVc as a template was carried out as described [29].

### Immunofluorescence

Standard immunofluorescence was performed as described previously [49]. Briefly, cells grown on glass coverslips were fixed for 20 min at room temperature with 4% paraformaldehyde, permeabilized using PBS+0.1% Triton-X100 during 4 min, rinsed with PBS, and blocked overnight at 4°C with PBS+2% normal goat serum. Incubation for 1 h at room temperature or overnight at 4°C with primary antibodies was followed, after several washes in PBS, by a 1-h incubation at room temperature with secondary antibodies. After extensive washing, coverslips were mounted by using Vectashield containing DAPI to stain nuclei (Vector Laboratories, Burlingame, CA).

### Stimulation of neuronal cultures, cell extracts and Western blot

Neurons were incubated for 60 min at 37°C in culture medium containing the blockers of neuronal activity TTX, APV and CNQX. Following this resting period, the medium was replaced by medium containing 1 µM PMA and neurons were stimulated for 10 min. Neurons were then rapidly washed in ice-cold PBS and harvested in lysis buffer containing phosphatase inhibitors [57]. The rest of the procedure was performed as described [49]. Briefly, equivalent amounts of cell lysates were separated by electrophoresis using 10% Bis-Tris Nu-PAGE gels (Invitrogen) and then transferred onto nitrocellulose membranes (Hybond-C extra, Amersham Biosciences, Orsay, France). After blocking (Li-Cor blocking buffer, ScienceTec, Les Ulis, France, or Tris buffer saline containing 5% non-fat dry milk), membranes were incubated with primary antibodies. Secondary fluorescent antibodies used were the following: IRDye 800CW goat anti-Mouse IgG (Li-Cor) or Alexa Fluor 680 goat anti-rabbit IgG (Invitrogen). Laser scanning and quantitative analyses of the blots were performed using the Odyssey Infrared Imaging System (Li-Cor). Quantification of protein phosphorylation was carried out by measuring the intensity of fluorescence of the band corresponding to the phosphorylated protein normalized by ß-tubulin expression, due to inefficient stripping of phospho-MARCKS and phospho-SNAP-25 antibodies binding. In parallel, total levels for each protein was verified on a separate blot. Results are expressed as percentage of increase over the mean of unstimulated controls, which was set to 100%.

### Free intra-cellular calcium measurements

Neurons grown on flat-bottom 96-well plates were incubated for 30 min at 37°C in HBSS (Invitrogen) –BSA (Sigma) solution containing 2.5 mM probenecid (Sigma), pH 7.45 supplemented with fluo-3 acetoxymethyl (AM) 1 mM (Invitrogen) and 20% pluronic F-17 (Sigma). After this incubation period, neurons were washed twice with HBSS-BSA-probenecid solution and placed into a 37°C incubator in the dark for 30 min. Fluorescence was measured at 460–490 nm excitation and 515 nm emission in each well, using a Novostar plate reader (BMG Labtech, Champigny s/Marne, France). For each well, a series of 250 recordings (one per second) was performed; neurons were first exposed to 50 mM KCl (Sigma) from 10 to 90 s, then to HBSS-BSA-probenecid solution containing 50 µM thapsigargin (Calbiochem, Fontenay-sous-Bois, France), 5 µM ionomycin (Calbiochem) and 10 µM EGTA (Sigma) from 90 to 177 s and finally to HBSS-BSA-probenecid solution containing 120 mM CaCl_2_ from 177 to 250s. [Ca2^+^]_i_ was calculated using the equation [Ca2^+^]_i_ = K_d_ (F−F_min_)/(F_max_−F), where K_d_ is the dissociation constant of the Ca^2+^-fluo-3 complex (390 nM), and F represents the fluorescence intensity of the cells expressed as the ratio between the highest fluorescence measurement between 10 and 90 s and the baseline. F_min_ corresponds to the minimum fluorescence between 90 and 177 s. F_max_ represents the maximum fluorescence between 177 and 250 s (see Figure 3A).

### Electrophysiology

Neuronal cortical cultures were prepared from embryonic Sprague Dawley rats at gestational day 18, according to a previously described protocol [37]. Neurons were seeded at a density of 10^5^ cells per MEA and half of the MEA dishes were infected with BDV on day 1. All experiments were made on day 21, to allow spreading of the different recombinant viruses to the totality of the MEA dishes. Signals corresponding to the electrical activity from the 60 electrodes of the MEA were recorded using the MC Rack Software (Multi Channel Systems GmbH, Reutlingen, Germany) for online visualization and raw data storage. The signal corresponding to the firing of a single action potential by a neuron in the vicinity of an electrode was identified as a spike. We also detected high frequency grouped spikes trains, known as bursts, which represent an important parameter of the analysis of neuronal network activity [58]. Spikes and bursts were detected by a dedicated analysis software developed at INSERM U862 (Bordeaux, France) [37], which computes the signal obtained from the electrodes, calculates a threshold and detects a spike every time the signal crosses this threshold with a negative slope. The threshold was set to a minimum of three standard deviations of the average noise amplitude computed over the whole recording and applied from the signal averaged value as a baseline [59]. Bursts were defined as a series of ≥3 spikes occurring in less than 100 ms. Measures were performed under spontaneous conditions, during a 15 min stimulation period using 50 µM Bicuculline, and after washout of bicuculline upon perfusing the MEA dish with 2.5 ml medium. After each manipulation, neurons were allowed to rest for 2 min before recordings were taken, to avoid vehicle effects. For each condition, recordings were performed over a 3 min period, and the mean burst frequency was calculated by averaging the results obtained for all electrodes.

### Data analysis

Data are presented as mean±standard error of the mean (s.e.m). Statistical significance was determined using Student's unpaired *t-*test.

### Accession numbers

The SwissProt (http://www.expasy.org/sprot/) accession numbers for proteins mentioned in the text are BDV Nucleoprotein (Q01552), BDV Phosphoprotein (P26668), BDV X protein (Q912Z9), BDV matrix protein (P52637), BDV polymerase (P52639), MARCKS (P30009), SNAP-25 (P60881), PKC epsilon (P09216), CKII (P19139), TBK-1 (Q9WUN2). The GenBank (http://www.ncbi.nlm.nih.gov/Genbank) accession number for the strain discussed in this paper is BDV strain He/80/FR (AJ311522).
